# Implementation of the free maternity services policy and its implications for health system governance in Kenya

**DOI:** 10.1136/bmjgh-2016-000249

**Published:** 2017-11-12

**Authors:** Thidar Pyone, Helen Smith, Nynke van den Broek

**Affiliations:** Centre for Maternal and Newborn Health, Liverpool School of Tropical Medicine, Liverpool, UK

**Keywords:** institutions, new institutional economics, health system and policy research, health financing policy, maternal health, qualitative study

## Abstract

**Introduction:**

To move towards universal health coverage, the government of Kenya introduced free maternity services in all public health facilities in June 2013. User fees are, however, important sources of income for health facilities and their removal has implications for the way in which health facilities are governed.

**Objective:**

To explore how implementation of Kenya’s financing policy has affected the way in which the rules governing health facilities are made, changed, monitored and enforced.

**Methods:**

Qualitative research was carried out using semistructured interviews with 39 key stakeholders from six counties in Kenya: 10 national level policy makers, 10 county level policy makers and 19 implementers at health facilities. Participants were purposively selected using maximum variation sampling. Data analysis was informed by the institutional analysis framework, in which governance is defined by the rules that distribute roles among key players and shape their actions, decisions and interactions.

**Results:**

Lack of clarity about the new policy (eg, it was unclear which services were free, leading to instances of service user exploitation), weak enforcement mechanisms (eg, delayed reimbursement to health facilities, which led to continued levying of service charges) and misaligned incentives (eg, the policy led to increased uptake of services thereby increasing the workload for health workers and health facilities losing control of their ability to generate and manage their own resources) led to weak policy implementation, further complicated by the concurrent devolution of the health system.

**Conclusion:**

The findings show the consequences of discrepancies between formal institutions and informal arrangements. In introducing new policies, policy makers should ensure that corresponding institutional (re)arrangements, enforcement mechanisms and incentives are aligned with the objectives of the implementers.

**Key questions**

**What is already known about this topic?**

- Reports on the introduction of the free maternity services (FMS) policy in Kenya describe either the implementation (including the perspectives of health workers) or health system outcomes (provision of free maternal health services or increase in use of the service).

**What are the new findings?**

- Our study explores how the FMS policy affected the way in which the rules governing health facilities are made, changed, monitored and enforced.
- Our analysis and interpretation was informed by the institutional framing of governance, in which governance is defined by the rules for distributing roles among key players and shaping their actions, decisions and interactions.
- To our knowledge, no other studies have examined implementation of the FMS policy and how the rules governing health facilities in Kenya have been enforced, monitored and changed.

**Recommendations for policy**

- The findings highlight discrepancies between formal institutions and informal arrangements, and their consequences. Therefore, in introducing new policies, policy makers should ensure that corresponding institutional (re)arrangements, enforcement mechanisms and incentives are aligned with the objectives of the implementers.

## Introduction

User fees are an important source of income for most health facilities in low- and middle-income countries,^1 2^ and introducing a health financing policy into a health system, particularly one which directly affects user fees, is not straightforward.^2 3^ Removal of user fees can have consequences for health facilities and the way they are governed. For example, the extent to which health facilities comply with the new policy (formal rule)—that is, choose to discontinue levying user charges, may be influenced by the extent to which the policy is in line with existing norms and practices at the health facilities (informal rules). Despite its importance, this rules-based approach to studying health system governance has not been widely adopted in health policy and systems research.^4^

**Figure 1:**
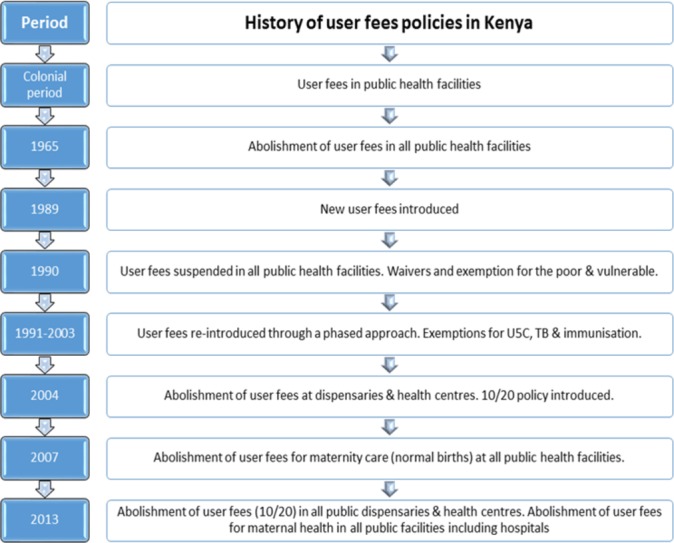
History of user fees policies in Kenya. 10/20 policy: ceiling on user fees of KSH10 (US$$0.10) in dispensaries and KSH20 (US$$0.20) in health centres

In Kenya, several policies to reduce the financial burden of healthcare have been introduced since independence in 1963 (figure 1). In 1996, the National Hospital Insurance Fund (NHIF) was established to finance healthcare in public and private facilities.^5^ This mandatory insurance scheme applies to workers from the formal sector earning more than KSH1000 a month. In 2004, the government set a ceiling on user fees of KSH10 (US$0.10) in dispensaries and KSH20 (US$0.20) in health centres (known as the 10/20 policy) with an exemption for children aged <5 years, the poor and those with designated conditions such as malaria and tuberculosis.

In 2007, in addition to these exemptions, all fees for maternity care (uncomplicated births) at public healthcare facilities were abolished. The government then introduced the Health Sector Services Fund (HSSF), which transferred funds directly to health facilities to compensate for the loss of revenue as a result of the removal of user fees. Financed using domestic and donor resources, the HSSF was officially launched in 2010 with the aim of pooling public and donor resources.^5^ The funds are locally managed by the health facility management committee, composed of community members.^6^

In 2010, the same year that the HSSF was launched, Kenya adopted a new constitution which devolved political, administrative and financial functions to 47 newly created administrative units known as counties. In the health system, this involved devolving essential health service delivery to county governments while the central government retained responsibilities for health policy development, management of provincial hospitals (now national referral hospitals) and technical support to counties. However, there are no clear guidelines on how to implement the HSSF in the newly devolved health system.^6^

In June 2013, the government introduced two additional policies to reduce the financial burden of access to healthcare: abolition of user fees for all health services in public healthcare centres and dispensaries (primary care level); and free maternity services (FMS) services at all levels of care in the government health sector (primary, secondary and tertiary).^7^ These policies, FMS in particular, were part of a national strategy to reduce maternal and neonatal mortality, alleviate poverty and achieve the millennium development goal targets.^7^ The FMS policy was introduced when the Kenyan health system was undergoing devolution.

The Kenyan health system is evolving and new financing policies are being introduced within the recently decentralised system. This study explores how the implementation of the FMS policy, introduced in 2013, has affected the way in which the rules governing health facilities are made, changed, monitored and enforced. The framework used in this study allowed us to highlight challenges to the implementation of FMS, particularly governance.

In this qualitative study, the experiences and opinions of stakeholders at the health facility, county and national levels were analysed with a focus on institutions. This focus reflected the new institutional economics approach in which institutions are defined as encompassing both the formal and informal rules that underlie social and economic activity.^8^

The application of institutional economics as a theoretical basis for health systems research is not new: Bertone and Meessen developed a framework to evaluate the implementation of performance-based financing in Burundi.^9^ Abimbola *et al* adapted the multilevel framework developed by Ostrom^10^ to assess governance of primary healthcare in Nigeria.^10–13^ Furthermore, Abimbola *et al* used the concept of transaction costs originally developed by Williamson (1979) to explore the barriers to accessing tuberculosis services in Nigeria.^14^ Other examples of institutional analysis can be found in our recent systematic review of frameworks for assessing health system governance.^15^

Previous reports and publications on introduction of FMS in Kenya describe either the implementation (including the perspectives of health workers) or health system outcomes (provision of free maternal health services or increased use of the service) without highlighting governance.^7 16–18^ In this study, we report an institutional analysis of the implementation of FMS policy in Kenya. The aim of this study is to understand how the policy altered health system governance in Kenya and to use the insights to inform policy implementation in Kenya and in other low- and middle-income countries.

## Methods

### Study settings

The study was conducted in six of the 47 counties which make up Kenya (Nairobi, Machakos, Makueni, Kitui, Nakuru, Narok) purposively selected for logistical and feasibility reasons. According to the Kenya Service Availability and Readiness Assessment Mapping Report of 2013, the counties included in the sample had a total population of 9.7 million (approximately 22% of the country).^19^ The six counties are served by 2066 health facilities, of which, 38% (784) are public health facilities (table 1).

**Table 1:** Key health indicators of the counties included in the study

Selected key indicators	National	Kitui county	Machakos county	Makueni county	Nairobi county	Nakuru county	Narok county
Literacy rate of women aged 15–49 years	87.8%	91.7%	95.3%	96.9%	96.5%	94%	94%
Delivery with skilled attendants	61.8%	46.2%	63.4%	54.6%	89.1%	69.5%	40.3%
Contraceptive prevalence rate	58%	57.3%	75.9%	80.3%	62.6%	56.8%	47.8%
All basic immunisation coverage aged 12–23 months’ old	79%	69.3%	93.4%	92%	81.2%	79.2%	66.4%

Source, KDHS (2014).

### Study participants

Three groups were invited to take part in the study: (1) national level policy makers and key international players, (2) county level policy makers and (3) policy implementers at healthcare facilities. Participants were purposively selected using maximum variation sampling. In total, 39 out of 43 people invited were interviewed: 10 national level policy makers, 10 county health officials and 19 healthcare providers from 10 purposively selected district and county level hospitals. Unavailability during the data collection period was the only reason given for non-participation. 

**Figure 2:**
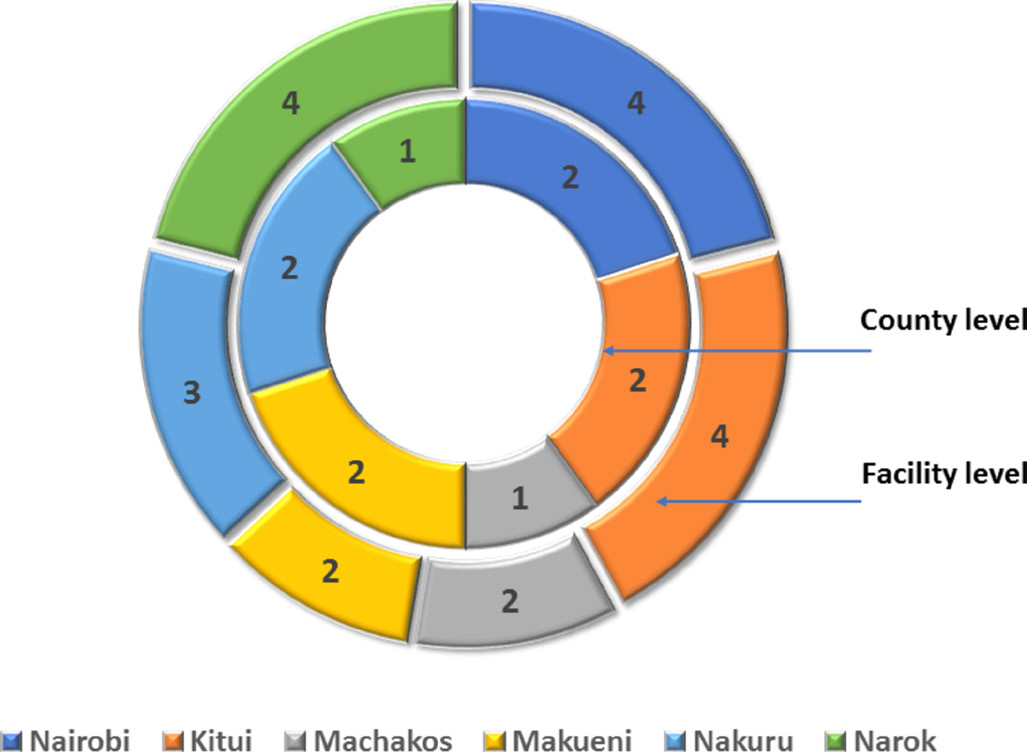
Distribution of county and facility level respondents who participated in the study.

Table 2 and figure 2 illustrate the characteristics of respondents who participated in the study and their roles in maternal health policy in Kenya.

**Table 2:** Characteristics of respondents who participated in the study

Health system level	Institution	Participant	Participated (n=39)	Invited (n=43)
National (policy)	Ministry of Health; multilateral and bilateral organisations	Senior directors and advisors	10	11
County (policy)	County Health Department	Chief Officer of Health	5	6
		County Director ofHealth	5	6
Facility (implementation)	Government health facilities offering comprehensive maternity care	Doctor in charge	10	10
				Nurse in charge of maternity	9	10

### Data collection and management

Data collection was carried out between April and October 2015. Semistructured interviews were conducted with a topic guide comprising a list of open-ended questions exploring factors influencing governance of the health system after the introduction of FMS in 2013. Topics discussed included: key strategic policies in maternal health including the FMS policy; stakeholder involvement in policy development; the extent to which the policies have been enforced and the responsiveness of these policies to the population’s needs. Depending on the availability and willingness of the respondents, interviews lasted between 30 and 90 min. All interviews were conducted in English, transcribed verbatim into Microsoft Word.

### Data analysis

**Figure 3:**
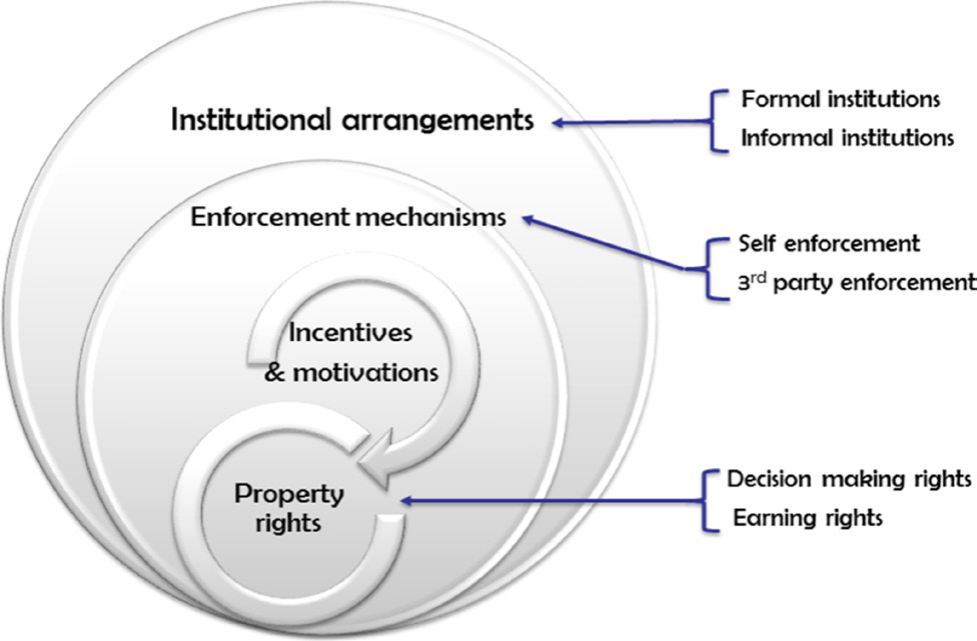
Illustration of the theoretical framework used in the study.

The approach taken was ‘directed content data analysis’, in which data are organised using an existing theoretical framework.^20^ Inductive coding was used to identify codes related to implementation of the FMS policy. Once the codes were identified, a deductive approach was used to group the data into relevant categories based on the theoretical framework adopted in this study.^8^ (figure 3)

### Theoretical framework

Douglas North defines institutions as ‘the rules of the game’ and organisations as ‘the players of the game’.^8^ Institutions consist of formal rules (political or legal rules such as constitutions, policies and contracts) and informal rules (such as code of conduct, behavioural norms and conventions).^8 21^ On the other hand, organisations comprise groups of individuals with common objectives.^8^ North’s principal argument is that the actions, decisions and relations of individuals within an organisation are both enabled and constrained by formal and informal rules which constitute the institutions.^8^

We used this framework and refer to health facilities (and the health workers who work within them) in this study as organisations while ‘institutional arrangements’ are the configurations of the rules. Enforcement of the rules^8 21^ can occur via self-enforcement, such as common beliefs, reputation, kinship, or via third-party enforcement, such as legal sanction, contracts, rules, laws or policing.^8^

Incentives and motivations are instrumental in achieving the organisational objective—for example, delivery of FMS. In line with the ‘principal-agent’ theory, agents (ie, health workers in a health facility) may comply with the FMS policy if incentives are aligned with the agents’ expectations.^22^ In this study, two types of motivation are discussed: intrinsic motivation (the health workers comply with the new policy arrangement as s/he values the goal of the policy) and extrinsic motivation (the health workers expect to benefit by complying with the new policy).^9^

Formal and informal rules and their enforcement mechanisms define ‘property rights’ of individuals. ‘Property rights’ include decision-making rights and earning rights which the individuals hold on different assets (eg, patent, trademark, a piece of land).^23^‘Decision-making right’ is the decision space or decision-making authority allowed by the principal (eg, the government) to agents (ie, health facilities).^24^ There are two forms of decision space: formal decision space allowed by formal rules and regulations which can be enforced, and informal decision space which is the actual exercise of authority which may deviate from the rules.^24^ In this study, ’earning rights’ refers to the decision space allowed to health facilities to generate income through user fees.

### Ethics

This study was approved by the Liverpool School of Tropical Medicine ethics review committee (ref no: 14–052) and the Kenyatta National Hospital-University of Nairobi ethics research committee (ref no: KNH-ERC/A/98). Participation in the study was voluntary and no incentives were given. At the beginning of each interview, the participants were asked to read the letter of consent, indicate their willingness to participate and record it on the consent form. The researchers also obtained consent to audio record the interview. Anonymity of study participants was assured and maintained during data management, analysis and dissemination.

## Findings

Using the theoretical framework adopted in this study, illustrated in figure 3, we identified the following four themes that highlight the ways in which the FMS policy affected health system governance: (1) formal and informal institutional (re)arrangements; (2) enforcement mechanisms; (3) property rights and (4) incentives and motivation.

### Institutional (re)arrangements

#### Formal institutional (re)arrangements

The president of Kenya abolished user fees for maternal health services in public facilities in June 2013. Subsequently, the national Ministry of Health (MOH) issued a circular confirming the abolition of user fees and provision of free maternal healthcare. Respondents across all participant groups (national, county and facility level) agreed with the objective of abolishing fees for maternity services. However, they observed that introducing the FMS policy at the same time as devolution affected implementation of FMS. In particular, they recognised that devolution had changed the way in which the health system was governed. For instance, responsibility for governance had been devolved from the national government to the county.

At health facility level, respondents thought that the FMS policy was too limited as it included only public health facilities (ie, less than half of healthcare facilities across Kenya) (box 1, KI-10). Respondents from the MOH also acknowledged these limitations. The government has subsequently been trying to find ways to integrate the policy with other financing mechanisms such as the NHIF, a scheme established to finance healthcare in both public and private facilities, and include private facilities under the FMS scheme.

Participants mentioned the institutional arrangements for reimbursement and the complexity of the process. Facilities must request funding through the county health department which, in turn, submits facility data (eg, the number of births) to support the claim to the National Health Treasury. The National Health Treasury verifies the county requests by triangulating the data received with routine District Health Information System data. Funds are then transferred to the relevant county health treasury where further processing takes place before the funds reach the health facility. Respondents at all levels of the health system had encountered challenges because of inefficiencies in the reimbursement processes, including delays in receiving disbursements and the receipt of insufficient funds. These challenges frequently affected planning at the health facility level (box 1, KI-16).

Informants from different levels of the health system highlighted the consequences of weak formal rules—for instance, the inefficiency of information dissemination, as they did not receive the correct information (the content of the FMS package and reimbursement amount) in time. Health workers had ‘*not seen a written circular and it was difficult (for them) to know what was free*’ (KI-9). Health workers explained their dilemma regarding the public expectation versus the actual content of the policy, *‘the public expects that when they walk through the (facility) gate, everything should be free*’ (KI-9). Some counties sometimes ‘*threatened to charge*’ user fees for maternity services while others continued to charge for the first few months after the FMS policy was introduced until the information and guidelines became clear (box 1, KI-15). National level respondents acknowledged the gap in information dissemination explaining that the level of refund was according to the level of facility and type of delivery (box 1, KI-5). In some facilities, the FMS package covered the continuum of care, including antenatal, delivery and postnatal care for up to 1 year, whereas elsewhere, the policy covered only delivery services. Others questioned whether complications during pregnancy, such as ectopic pregnancy and abortion, should be included as part of the policy, others mentioned charging a woman who returned to the facility with complications after she had been discharged.

The information gap was also seen between the county government and health facilities. Health workers were not satisfied with the arrangement as they did not receive information about reimbursement and some wondered whether ‘*the county uses it (the funds) without consulting them*’ (KI-36). Information was not shared among the policy implementers or with the clients, who were also confused by the changes in health financing. The facility service charter was displayed in some facilities, providing information to clients about the services for which they were required to pay. However, participants also gave examples of health workers or casual staff taking advantage of the lack of clarity by asking women for payments for maternity care in accordance with the previous user fee system (box 1, KI-25, 24, 19).

Box 1 **Illustrative quotes: formal institutional arrangements**

“If we are taking care of 50% of the healthcare, then why not have half the board from faith based and the other half from the Ministry.” (Health worker, KI-10)

“You cannot give everything for free…and if the government abolishes user fee, then they must support the hospitals and give them money to run, but as it is now, money does not come. XX hospital received money only once and it was last year in January, since 2013 when the President started this policy he has never given (our hospital) any money and maybe it is not his fault, maybe he doesn’t even know, but the people below him are sitting on that money.” (Health worker, KI-16)

“So, there was no understanding and some places even right now I read in the newspaper, that some counties were going back to, were threatening to start charging and actually some facilities were charging hospitals. But why they were charging is because of the government. They were complaining that the government is not reimbursing promptly.” (National policy maker, KI-15)

“Money was being delayed. Because it was the facility to provide the services and then they get reimbursement after providing us with the data or of the women were delivered. But you know, get those reports and all those things are usually lengthy and some delayed.” (National policy maker, KI-5)

“Last year I had an incidence and we took it up to the office. It was a casual worker, in the evening, she was going around and telling them ‘*Have you been discharged? You want to go home tomorrow give me money*’. Actually, it was two thousand at that time and the mother was a bit in doubt, why has she asked for money and these are free services, so she kept quiet and called the nurse later.” (Health worker, KI-25)

“Give me five hundred shillings, I sell to you, the things could be in theatre, they could be available, but now because the mother is desperate, the relative wants his or her patient to be operated on, now someone takes advantage…. they thought the surgical spirit or the betadine, they thought it is not available in the hospital and they need to go and buy so that the patients are operated on while the things are still there this guy just wants to exploit.” (Health worker, KI-24)

“You find patient has not been given a receipt then you ask the patient and find the person who was there, took the money and did not give the receipt, so those are the people we deal with most of the time, petty issues like taking money from patients.” (Health worker, KI-19)

#### Informal institutional (re)arrangements

It became obvious during the interviews that the newly introduced formal institutional (re)arrangements were unclear and posed important challenges for implementation. To overcome these challenges, county officials and health workers (ie, policy implementers) introduced informal institutional (re)arrangements. For example, some suggested it was necessary to divert funds from the county budget to pay for services provided under the new rules *‘while waiting for reimbursement*’ under the FMS policy (box 2, KI-30). County health officials also encouraged health facilities to make use of the NHIF to alleviate the burden of the poor reimbursement processes under the new policy—that is to ask women participating in the NHIF scheme to cover the costs of maternity care using their insurance scheme requiring out-of-pocket co-payments. But the women were reluctant to pay for maternity care using their insurance as they assumed the services were free for all (box 2, KI-39).

Another informal arrangement to ensure the availability of drugs and supplies occurred as a result of reimbursement problems, which inevitably meant charging women some fees. Health facility staff admitted that they had to write prescriptions for women as the facilities frequently had shortages of medicines and supplies (box 2, KI-11). In cases of severe shortages, health workers asked women to buy all consumables and only the consultation itself was free (box 2, KI-36). Another informal arrangement used by staff at facility and county level to overcome shortages in medicines and supplies due to late reimbursement was to ‘borrow’ from all other suppliers. Participants explained how they used this arrangement to ‘cope’ with the situation. Suppliers were reimbursed when payments were received from central government (box 2, KI-21).

Every county level respondent who participated in the interviews described how they had had to make their own arrangements to overcome the challenges faced in implementing the FMS policy. Of particular concern was the effect of the policy on the workload of existing staff due to the increased numbers of women attending services. Participants described making their own arrangements to hire locums or postgraduate doctors (whose over-time work was not paid) to deal with the shortage of medical doctors, and part-time nurses or student nurses to ensure sufficient nursing staff (box 2, KI-22, 25).

Box 2 **Illustrative quotes: informal institutional arrangements**

“We have been funding some of these programme on the county budget, for example when you look at the referral we have been funding from the county budget as we wait for the funding from the maternity reimbursement.” (County official, KI-30)

“They are using the NHIF and maybe they are given the prescription to buy the drugs., It is a challenge because somebody tells you I am using the NHIF card and you are telling us to buy the drugs, so it is hard to convince that person to tell him to go and buy the drugs. But at times, they used to buy because there is nothing else you can do.” (Health worker, KI-39)

“Even you go to XX facility, you find that most of them will come and go there, they don’t get medications…, it has to be free but it’s not fully free. They have to pay some fee.” (Health worker, KI-11)

“Actually, we tell them to buy everything…we should have just told them to pay and use that money (ourselves at facility level) to buy whatever we need. It could have been better than us telling them it is free and (then) we are not able to provide them good quality services.” (Health worker, KI-36)

“You have to borrow from the suppliers and promise to pay when you get the money, so that is how we have been coping.” (County official, KI-21)

“We have so many mothers, it (the FMS) is a good policy, I think it’s in the right place, but they need to increase now the number of personnel to take care of these people because it can be overwhelming.” (Health worker, KI-22)

“But my particular facility, it hosts student nurses, so some procedures under supervision they can ease the work load.” (Health worker, KI-25)

### Enforcement mechanisms

When built-in formal enforcement and self-enforcement mechanisms failed, mainly because county and health facility stakeholders felt excluded from the policy design, only third-party enforcement mechanisms seemed to be effective. For instance, participants at facility level talked about the media as important enforcers, acting as watchdogs for health facility accountability. The staff at some facilities were concerned about the potential loss of reputation and scathing media reports if they were found to be charging for maternity services (box 3, KI-16).

Respondents at county and facility level believed that there would have been more self-enforcement and better co-operation by implementers if they had been invited to participate in the development of the FMS policy. Some county level respondents said that third-party enforcement mechanisms were therefore essential under these conditions. Others commented that facilities in hard to reach areas where supervision mechanisms were weak required more stringent third-party enforcement through *‘impromptu supervision*’ in addition to ‘*scheduled supervision*’ to ensure the FMS policy has been implemented properly (box 3, KI-26).

Efficient third-party enforcement mechanisms could prevent potential embezzlement and ensure health workers do not take advantage of the situation, particularly the information asymmetry. Facility level respondents shared experiences of exploitative practices—for example, casual workers and theatre staff who asked for payment from women using outdated payment rates from the previous user fees system and did not provide receipts.

Box 3 **Illustrative quotes: enforcement mechanisms**

“We cannot charge because we are in Nairobi and if we charge there will be a lot of noises. They will know immediately, the media will come immediately. So, we cannot charge, so we just run the hospital with the little we have until (maybe) it closes.” (Health worker, KI-16)

“The rural facilities sometimes are a bit distant we cannot monitor them on a day-to-day basis, so once in a while you will have a mother coming to deliver and they are asked to pay… we are aware that happens in some areas, but the way to avoid that or the way to mitigate that is to have supervision… they have scheduled supervision visits to those facilities that is one. They also have impromptu supervisory visits at the facilities.” (County official, KI-26)

### Property rights

The new partially enforced formal institutions and the informal institutions, which key stakeholders introduced to help them cope, redefined the property rights (both decision-making and earning rights) of health workers. For instance, previously healthcare facilities held the HSSF in their own bank accounts, allowing them to manage their own budget. In the devolved system, budgets are now managed by the county health treasury, which allocates funds to healthcare facilities within their catchment area. Income generated at the health facilities is therefore transferred to the county as county revenue. Under the current devolved system, facilities have lost the right to manage their own budget, including the procurement of medicines, supplies and equipment. Some healthcare facilities have closed their HSSF accounts. Some health workers perceived the new set-up as ‘*centralisation within a decentralised system*’ while others felt that ‘*everything is meant to be done at the county level*’.

### Incentive and motivation

The property rights of health workers diminished as a source of funding was lost and compensation for this was insufficient, with consequences for the supply of drugs and informal charges to patients. This means that incentives are not well aligned between principals (the MOH) and agents (health workers). This results in a loss of motivation among health workers and, in turn, poorer performance, as well as a series of critical negative consequences for the health system (box 4, KI-7,11,38,38).

Almost every respondent recognised that the FMS policy had increased the number of women receiving skilled birth attendance in their area (box 4). As a result, they appeared motivated as they had contributed to improving maternal and neonatal health outcomes (box 4, KI-35). At the same time, health workers perceived the rearrangement of the HSSF as demotivating, as health facilities lost their decision-making power and were no longer able to manage their own funds (box 4, KI-15,18). This situation was compounded by uncertainty about the availability of funds as they depended on the budget allocated to them by the county health treasury. Facilities could not plan properly owing to the irregularity of the disbursements and unpredictability of the amount received. Even respondents at county level acknowledged that, at times, the disbursement amount did not correlate with the numbers of deliveries conducted and there was no rationale for the value of the disbursement received, leaving both facilities and counties confused (box 4, KI-31).

Owing to the poor implementation of the FMS policy, there were few incentives to comply and it met with resistance at both county and health facility levels. Some were concerned with the challenges they faced every day in implementing the policy while others felt uncertain owing to a lack of clarity about the component of services under the FMS package. Additionally, some health workers did not see any obvious incentives for them as they were already stretched by their current workload (box 4, KI-9, KI-10). Some healthcare providers complained that implementation of the FMS policy had doubled their workload with no corresponding increase in human resources for health. National level respondents were aware of this, but believed that the human resource shortage was worse in those parts of the country where there were problems with conflict and security (box 4, KI-15).

Respondents described how the policy did not set correct incentives for health workers and reduced their motivation. This led to a loss in quality of care which had an opposite effect to that intended by the policy (box 4, KI-2, KI-15). Additionally, some health workers reported that the community felt that they received less attention from health workers when they accessed care due to the increase in the number of patients at the health facility (box 4, KI-38).

If stakeholders in charge of implementing the policy (counties and health workers) have doubts about its impact and sustainability, the policy will not influence their intrinsic motivation to improve the health of the population (box 4, KI-26). This, coupled with the decreased extrinsic motivation (ie, the policy does not provide financial incentives for them to implement it and expand the quantity and quality of care), shows how the policy struggles to work in practice. Health workers also remarked that the policy makers did not understand the practical, on-the-ground situation and hence did not anticipate or appreciate the operational challenges (box 4, KI-32, KI-10).

Box 4 **Illustrative quotes: incentives and motivations**

“…even if you provide maternity free services as a policy you need human resource and right attitude, a motivated one to provide the services for you to see that the indicators are good. So that aspect of motivation, in terms of taking care of the health worker, has not been streamlined, you get it?” (County official, KI-7)

“Some of the healthcare workers in some of the institutions because of lack of motivation, lack of good pay and nowadays we go slow.” (Health worker, KI-11)

“…not just motivation but you find that there could be a number of issues that you would want to try and be supportive…because maybe their salary is delayed for some time and they need to have a stable source of generating income.” (Health worker, KI-38)

“Yes, there are consequences. We had a mortality, a maternal mortality of which an audit, our conclusion was the system failed the patient and not the other way around…this was a patient who followed everything as was advised but the system failed her. She came to the hospital, she had a low Hb, we talked to the lab who told us they had blood, so we started transfusing. Other patients came who needed blood. Those patients were given the blood then blood was over. They did not inform us, we wanted three pints because one pint would sustain the patient. The patient went into labour. We had a black out the generator was not working. So, the patient delivered in the dark, the placenta was not completely out. The person who was taking care of the patient at that point did not know, because they were using their own torch, but other than that when the patient kept bleeding. This person kept on doing what she thought was supposed to be done which was half right because they did not know exactly what they were supposed to do. So, at that point when I was called in to see the patient we needed blood. There was no blood. We needed a vehicle to refer the patient; there was no fuel. We had just got a new medical superintendent, the nearest petrol station where we get the fuel they did not know this new medical superintendent. So, they were like they don’t know who they are dealing with and they will not give free petrol and basically, we were resuscitating a patient we know was dying, so there was nothing we could do.” (Health worker, KI-38)

“…initially they used to pay and because of lack of funding, then more mothers are not likely able to come and deliver and deliver from skilled attendants, so but with the introduction of free maternity, we are seeing now most of our mothers are coming to maternity places and of course that goes along in trying actually taking care of the reducing maternal and neonatal death and getting services from our trained personnel.” (County official, KI-35)

“…at the facility level, you have not received what you requested, because the management works with the devolution, some of the facilities accounts were closed what they used to earn, so it is being managed at the county level… the hospital management fund where you collect your revenue bank on a quarterly basis…” (National policy maker, KI-15)

“…initially the facility used to have the capacity to budget and spend facility improvements fund through the facility improvement fund, but under the devolved government, procurement is done from the county and the hospital no longer have a hospital account.” (Health worker, KI-18)

“The other challenge has been in terms of a capture of data, the facilities give their data of the deliveries they conducted, when they look at what they are compensated, at times they (the central government) don’t mention the month so it is very hard to go back and audit and see.” (County official, KI-31)

“Free maternity has increased the number of patients for us and human resource is a challenge. It means the demands are higher but the staff they are low.” (Health worker, KI-9)

“Because you have suddenly your maternity is full but you have the same number of nurses working … so even the quality of care is reduced.” (Health worker, KI-10)

“We have about 40% increase in terms of the workload but the number of health workers… some counties yes they are reporting that they have employed more but still below the expected numbers…I believe this is worse in some of the North-eastern regions where there has been conflicts or the terrorist threats so people have moved.” (National policy maker, KI-15)

“The challenges are if I go to a facility, I am not asked to pay but if you are putting me on the floor to deliver because there is no bed or there is no privacy, their curtain is all wide open, I may not use those services.” (National policy maker, KI-2)

“Removing that (financial) barrier, many people clog the facilities so the health workers were overwhelmed, quality is compromised so some of the regions refused to go to that free, they said they’d rather pay and get that quality service.” (National policy maker, KI-15)

“When you get to ask some of them, they would be like you increased, you encouraged us to come to hospital, you eliminated the fees that maybe would prevent us from coming, but my friends who came or my neighbours or my relatives who came and they said there was no one really to take care of them in the hospital.” (Health worker, KI-38)

“We have started experiencing that because in this county for example for the past 1 year, we have delivered mothers and we have not been reimbursed, one hospital like XX hospital which is very busy is owed around 20 million by the national government, so that I think is a challenge because this thing of just giving money, giving money and the resource purse is very limited might not be sustainable.” (County official, KI-26)

“The way they were intended is not exactly what is happening on the ground. They were supposed to be provided with free antenatal services, free delivery services, free post delivery services up to 6 weeks and free contraceptives after delivery, but when you go to what is happening on the ground, some facilities offer them free but they are not many. Most facilities still charge for everything except the delivery process itself, so at the end of the day it is not as free as it will appear to look.” (Health worker, KI-32)

“The President just woke up one day, in one of the National Celebrations, he decided there is free maternity care.” (Health worker, KI-10)

## Discussion

This study highlights the consequences of discrepancies between formal institutions and informal arrangements when a new financing policy on health system governance is intoduced. The findings point to misalignment of incentives for policy implementation, which was further complicated by the concurrent devolution of the health system. Efforts to enforce the policy implementation include self- and third-party enforcement, but there were significant disincentives for health system personnel at the county and facility levels to adhere to the policy. In many instances, implementation of the FMS policy was compromised by operational challenges, including delay in receiving reimbursements at health facilities. The policy also exacerbated existing health system weaknesses, such as the shortages of health workers, drugs and supplies.

This study reveals ‘*policy-induced institutional incoherence*’ because of the discrepancies between formal and informal institutions. A similar example was reported in Niger when a free primary healthcare policy was introduced for children aged <5 years in 2006.^25^ The policy reportedly weakened the functioning health insurance mechanism. Health workers in the public health sector had no incentive to adhere to the new rules, as they lost a reliable source of non-salary income. On the other hand, Booth^26^ described successful institutional coherence in Rwanda when the country introduced a new health insurance policy. The reform incentives were consistent with clear mandates from all line ministries and intense political pressure to comply with the reform.^26^ Those institutional (re)arrangements facilitated the reform. However, institutional coherence in introducing the FMS policy was not found in this study.

Consequently, implementers (counties and facilities) faced challenges of accountability, especially adherence to the FMS policy. For example, health workers who participated in the interviews openly admitted having to write prescriptions in order for clients to purchase medicines, including those covered under the FMS scheme. This finding is in line with the results of the evaluation conducted by the Kenyan MOH in 2014, with an estimated 28% of the women making some payment despite the free maternity services policy.^7^ Additionally, the Public Expenditure Tracking Survey (PET-PLUS 2012) reported that, on average, health centres charge KSH181 (US$1.81) for a normal delivery while dispensaries charge KSH45 (US$0.45).^1^

When resources are constrained, health workers are less likely to be accountable as they are not provided with the resources to work.^16^ This is shown by examples of non-adherence and exploitation of the situation provided by some health workers. Similarly, Cleary *et al* highlight three factors influencing the accountability of healthcare providers: (1) resources and capacity of key stakeholders; (2) attitude and perception of health system personnel and (3) values, attitudes and culture towards accountability mechanisms.^27^ Participants also expressed their concerns about the poor quality of services in overcrowded health facilities; this is particularly relevant for communities which cannot afford to seek care from other healthcare providers (ie, the private sector).

This study also highlights the way in which accountability has been compromised as the policy indirectly provides opportunities for corruption as described under ‘formal institutional arrangements’, where participants gave examples of health workers taking advantage of the situation. The outcomes of a system depend on how these rules are enforced and is in line with Gustafsson as, ‘*institutions without enforcement are not institutions at all*’.^28^ The introduction of an institutional delivery initiative in Rwanda was successful owing to rigorous enforcement of the rules, as the Rwandan government used a combination of financial penalty and social mobilisation to achieve public compliance with their new rule.^26 28^ Those activities were complemented by disciplinary actions against staff who engaged in abusive practices towards women.^26^ These complementary efforts, institutional arrangements being enforced and sanctions which were backed up by the political pressure, contributed to the fruitful and widespread adoption of institutional delivery in Rwanda.

Incentives to adhere to the new policy are helpful but the situation in Kenya placed pressure on health workers and inadvertently provided opportunities for abuses.^29^ The policy indirectly penalised health workers by not compensating for fees lost by the health facilities, pushing health workers to raise those revenues from patients. Similar findings were observed among Nigerian government officials working in the routine immunisation programme.^30^ The authors highlighted two motivating factors: incentives for high performers and a supportive working environment. Erchick *et al* emphasised that simply ensuring the basic needs of health workers (receiving sufficient salary on time) was crucial for motivating them to carry out their responsibilities.^30^

This study also highlights the importance of transparency and access to information at the right time. Respondents at all levels reported gaps in policy dissemination and information asymmetry. Similar findings were reported in the evaluation conducted by the Kenyan MOH in 2014, which reported that a circular describing the services included in the FMS policy was issued more than a year after initiating the policy.^7^ Finally, many of the key stakeholders were disappointed that they were not invited to participate in, or benefit from, the development of the policy. As they were not consulted during its development, stakeholders were unwilling to show commitment or comply once FMS became policy. Similar findings were presented in the Kenyan MOH evaluation report and elsewhere in sub-Saharan Africa.^7 16–18 30–32^

This study has a number of potential limitations. First, participants might have been reluctant to openly express their opinions during interviews for fear of recrimination. However, the researchers were independent of the local health system and this was reiterated at the beginning of each interview. In addition, the research was conducted at just one time and implementation of the policy might have differed in the counties excluded. Nevertheless, the six sampled counties comprise approximately 22% of the country and information from different respondents and the literature was triangulated to minimise bias. This study was conducted 2 years after introduction of the FMS policy; hence, it is impossible to distinguish the effects of FMS from those of other policies. The FMS policy was not introduced in a vacuum as other user fee exemptions existed; organisations had already, to some extent, adjusted to this. We limited our study to the FMS policy, but recognise that other exemptions and user fee policies exist and to some extent will have already modified, or had implications for, the health system and governance. However, we focused the interviews on the specific FMS policy introduced in 2013.

For the new institutional arrangements to be implemented successfully, organisations and organisational practices are crucial. This study highlights how governance of a health system was compromised while implementing a specific health policy to achieve a health system objective (universal health coverage). Our study provides useful insights for policy makers into what works and what does not work when introducing a new set of policy initiatives. This study reminds policy makers that it is critical to review and adjust the implementation of a new rule/policy in line with the organisational capacity (health system capacity) to implement.^33^ One of the main factors affecting compliance with the policy was the shortages of medicines and supplies. This echoes the results from other studies in Kenya and elsewhere where the removal of user fees was not accompanied by other reforms necessary for implementation.^17 18 25 32^ Hence, implementers lack the incentives to comply with the policy as they encounter operational challenges and perceive implementation as not feasible. Lack of participation in policy development is another reason for non-compliance. A bottom-up approach to implementing a given policy is essential to ensure that health workers, facility management committees and county health management teams are involved throughout the process.

We recommend improving mechanisms to ensure timely reimbursement of free services to facilitate the successful implementation of a new policy. Policy makers should undertake a detailed examination of FMS policy elements and specify what needs to be reformulated to strengthen the logical link with the ultimate aim of universal health coverage. We also recommend effective dissemination of information and raising of public awareness to facilitate compliance with the policy. Institutional arrangements can be strengthened by strict enforcement mechanisms and application of sanctions. Finally, this study recommends establishing incentives and ensuring that appropriate enforcement mechanisms are in place to ensure successful implementation of a new rule/policy. For instance, incentives can be established by ensuring regular and timely disbursements (including FMS reimbursements and health workers’ salaries) and adequate supplies of drugs to comply with the new rule/policy.

## Conclusion

Our study explores the implications of the implementation of the FMS policy on health system governance using institutional analysis as a theoretical framework. To our knowledge, no other studies have examined how implementation of the FMS policy has affected rules governing health facilities. The study highlights the discrepancies between formal and informal rules which create a misalignment of incentives for policy implementation. It is crucial to introduce new policies with corresponding institutional (re)arrangements, enforcement mechanisms and incentives aligned with the objectives of the implementers. It is critical for policy makers to review and adjust the implementation of a new policy, including consideration of organisational capacity and implementation processes. A more careful evaluation of the process is needed at the same time as policy implementation, with research designs to capture the processes of institutional change.
